# Assessing violence risk in first-episode psychosis: external validation, updating and net benefit of a prediction tool (OxMIV)

**DOI:** 10.1136/bmjment-2022-300634

**Published:** 2023-06-14

**Authors:** Daniel Whiting, Sue Mallett, Belinda Lennox, Seena Fazel

**Affiliations:** 1 Institute of Mental Health, University of Nottingham, Nottingham, UK; 2 Department of Psychiatry, University of Oxford, Oxford, UK; 3 Centre for Medical Imaging, University College London, London, UK; 4 Oxford Health NHS Foundation Trust, Oxford, UK

**Keywords:** Adult psychiatry, Forensic psychiatry, Schizophrenia & psychotic disorders

## Abstract

**Background:**

Violence perpetration is a key outcome to prevent for an important subgroup of individuals presenting to mental health services, including early intervention in psychosis (EIP) services. Needs and risks are typically assessed without structured methods, which could facilitate consistency and accuracy. Prediction tools, such as OxMIV (Oxford Mental Illness and Violence tool), could provide a structured risk stratification approach, but require external validation in clinical settings.

**Objectives:**

We aimed to validate and update OxMIV in first-episode psychosis and consider its benefit as a complement to clinical assessment.

**Methods:**

A retrospective cohort of individuals assessed in two UK EIP services was included. Electronic health records were used to extract predictors and risk judgements made by assessing clinicians. Outcome data involved police and healthcare records for violence perpetration in the 12 months post-assessment.

**Findings:**

Of 1145 individuals presenting to EIP services, 131 (11%) perpetrated violence during the 12 month follow-up. OxMIV showed good discrimination (area under the curve 0.75, 95% CI 0.71 to 0.80). Calibration-in-the-large was also good after updating the model constant. Using a 10% cut-off, sensitivity was 71% (95% CI 63% to 80%), specificity 66% (63% to 69%), positive predictive value 22% (19% to 24%) and negative predictive value 95% (93% to 96%). In contrast, clinical judgement sensitivity was 40% and specificity 89%. Decision curve analysis showed net benefit of OxMIV over comparison approaches.

**Conclusions:**

OxMIV performed well in this real-world validation, with improved sensitivity compared with unstructured assessments.

**Clinical implications:**

Structured tools to assess violence risk, such as OxMIV, have potential in first-episode psychosis to support a stratified approach to allocating non-harmful interventions to individuals who may benefit from the largest absolute risk reduction.

**WHAT IS ALREADY KNOWN ON THIS TOPIC**

- Violence perpetration is an important adverse outcome for some individuals presenting to psychiatric early intervention services. A prediction tool for violence (OxMIV or Oxford Mental Illness and Violence) provides a scalable way of improving consistency, raising the ceiling of expertise and risk stratification, but needs external validation in a clinical setting.

**WHAT THIS STUDY ADDS**

- This was the first external validation of a violence prediction model in a real-world clinical setting of early psychosis services. We reported prevalence of 11% violence perpetration in 12 months after first assessment. OxMIV discriminated well between individuals who did and did not perpetrate violence, and we recalibrated the model following a protocol. The prediction model was more sensitive than unstructured clinical judgement, which supports its use from a population health perspective.

**HOW THIS STUDY MIGHT AFFECT RESEARCH, PRACTICE OR POLICY**

- We have provided an approach to test a prediction model in mental health services, highlighting the importance of testing a range of performance measures, including calibration, using routinely collected data for external validations, and comparing the model with unstructured clinical approaches. The role of prediction tools, such as OxMIV, to prevent adverse outcomes needs more work on how it can be linked with treatments.

## Background

Schizophrenia spectrum disorders (SSDs) are associated with a range of adverse outcomes,^1 2^ which for some individuals can include violence perpetration. Violence is a leading cause of premature mortality and morbidity worldwide and a key target for public health interventions.^3^ Increased risk of violence perpetration in SSDs compared with unaffected control groups (including unaffected siblings) is robustly replicated in multiple observational studies using different diagnostic methods and outcome measures, with the important context that fewer than 1 in 4 men and 1 in 20 women with a SSD will perpetrate violence.^4^ However, for the minority where perpetration is a risk, implications for individuals, victims, families, clinical services and society (including economic impact^5^) can be substantial.

Preventative approaches can usefully focus on the early stage of illness, highlighted as a period of higher risk of violence perpetration which has a prevalence of around 10% in first-episode psychosis (FEP).^6 7^ Violence is associated with longer, more frequent hospital admissions, poorer function and victimisation,^8^ which underscores the importance of prevention.

The prognostic value of targeting assessment and intervention early in illness gave rise to specialist services, such as early intervention in psychosis (EIP) services in the UK, Australia, and Europe, and also Coordinated Specialty Care in the USA. The most recent evidence finds that these services reduce adverse outcomes, such as suicide and hospital admission.^9^ Early identification of needs around violence prevention would therefore align with an established focus on prognosis in FEP.

One approach to risk reduction, employed at scale elsewhere in medicine, is using prediction tools to support stratified interventions. Prominent examples are the Framingham Risk Score^10^ and QRISK calculator^11^ in cardiovascular medicine, which estimate individual risk to inform treatment decisions. Although no prediction tools in psychiatry are in widespread use, their potential application to psychiatric outcomes and settings is a key challenge,^12^ and could efficiently and consistently translate epidemiological knowledge into clinical practice.

For clinical impact, however, a prediction tool must be both accurate and clinically usable. Currently, only two violence risk tools have been studied in individuals with psychosis^13^: Historical Clinical Risk Management-20 (HCR-20^14^; a 20-domain instrument that prompts clinicians to make low/medium/high categories without probability scores) and Violence Risk Appraisal Guide (VRAG^15^; an actuarial tool of 12 numerically rated items including aspects of a psychometric tool (psychopathy checklist)). These are more typically used in forensic mental health populations due to their length (taking some hours for first completion), direct costs and lack of external validation in non-forensic settings. A new, scalable tool called OxMIV^16^ (Oxford Mental Illness and Violence) is a potential candidate for use in EIP services. It was developed and validated in individuals with schizophrenia spectrum and bipolar disorders in Sweden, with a focus on clinically available predictors.^16^ It performed well when tested in a separate geographical subset of data (with a c-index, equivalent to area under the curve (AUC) of 0.89).^16^ The model is transparently reported and freely available as a calculator with 15 predictors rated categorically (eg, sex at birth) and one continuously (age). Each item is weighted by a coefficient and combined with a constant in a manner that can be transferred to electronic health record (EHR) systems. It estimates risk of a violent offence in the subsequent 12 months in percentage and categorical forms (low/increased) and can be completed in minutes during a full clinical assessment.

Examining the transportability of OxMIV to EIP services through external validation is a key step toward clinical integration. This phase of prediction model research is often neglected^17 18^ but is necessary for translation into practice. This includes calibration,^19^ and if necessary, updating models to reflect different baseline risks in new settings.

## Objective

The aims of this study were to (1) examine the performance of OxMIV for predicting violence in individuals assessed by EIP services, with emphasis on clinical applicability, (2) update and recalibrate the model as necessary, and (3) compare the accuracy of OxMIV with that of current practice which is unstructured clinical assessment, to consider its role and benefit.

## Methods

### Study design and data sources

The study used EHR data from a retrospective cohort of individuals consecutively assessed by EIP services in two English counties between 2012 and 2018, combined with police data on violent occurrences. A protocol and Transparent Reporting of a multivariable prediction model for Individual Prognosis Or Diagnosis (TRIPOD) guidelines^20^ were followed (online supplemental table 1). Data were collected retrospectively, offline from the clinical setting, to ensure clinical decision-making was not affected. This is a necessary and distinct phase preceding any future trial of the tool’s impact, and allows essential parameters required for calculating a sample size for a full trial to be estimated.

10.1136/bmjment-2022-300634.supp1 Supplementary data

The included population was all individuals aged 14–65 years who received an in-person assessment with EIP services for Oxfordshire and Buckinghamshire.^21^ We included all individuals assessed, rather than only those fulfilling FEP criteria, to validate the model in a clinically useful way, as there is often diagnostic uncertainty at this juncture. To validate the model for use at the point of assessment, only information that would be potentially available at that assessment time can be used. This precludes, therefore, incorporating or selecting based on diagnostic information available at a later point. Further, the service must consider risk even for those not meeting FEP criteria, particularly as many will be referred to other mental health services.^6^ Individuals whose referrals were triaged but did not have full in-person assessment were not included.

Data to populate OxMIV were extracted from routine assessment documentation using a manual approach to increase the yield of information.^6^ Whether or not an individual was identified as at risk of perpetrating violence in this original clinical assessment was recorded, along with any significant violent incident in the subsequent 12 months.

### Definition of violent outcomes

Violence was a binary outcome defined as a police occurrence for a violent offence (online supplemental table 2), or an incident documented in the EHR of interpersonal violence involving a weapon or injury (regardless of whether this resulted in police contact). Injury was defined as documentation indicating physical sequelae, for example, bleeding, bruising and hospital attendance. Outcome data collection was blinded to the OxMIV score. Combining police and EHR outcomes avoided missing outcomes in individuals no longer in contact with mental health services (potentially due to, for example, incarceration following violence), and also avoided missing significant violence which did not result in police contact, such as during inpatient admission (online supplemental method 1).

### Definition of predictors

In model development, predictors were defined by coding in Swedish registers.^16^ Operationalised definitions were used for external validation (online supplemental table 3) to validate clinically usable definitions from the EHR.

### Clinical judgement comparator

Whether violence risk was identified in the original clinical assessment was recorded by examining the risk summary or medical correspondence closest to assessment, extracted in a binary manner using prespecified definitions (online supplemental methods 1).

### Sample size

Sample size was determined by the recommendation that, in external validation, a minimum effective sample size of 100 participants with and 100 without the event is required.^22^ A 10% event rate was estimated from previous literature.^6^ Based on an estimate that 85% of referrals result in face-to-face assessment, 1300 consecutive referrals were examined.

### Missing data

The proportion and pattern of missingness were examined. Multiple imputation by chained equations^23^ was then used to create 20 imputed datasets. This is the standard approach recommended in current statistical literature.^20 24^ Performance measures were calculated separately for each imputed dataset and pooled according to Rubin’s Rules^25^ (online supplemental methods 1).

### Model performance

Performance was examined with measures of discrimination, calibration and overall performance (see online supplemental methods 2 and online supplemental methods 3 for details, including decision curve analysis methods and model updating). For the updated model, paired measures of misclassification around a decision threshold (sensitivity, specificity, negative predictive value (NPV) and positive predictive value (PPV)) were calculated for a range of clinically sensible cut-offs. This can assist in contextualising accuracy for potential linked decisions and for interpreting performance in comparison with the data on binary clinical judgements.

## Findings

### Included population

Of 1300 individuals consecutively referred to EIP services, 1145 received an in-person assessment (online supplemental figure 1). Of all 1145 assessed, 131 (11%, 95% CI by Wilson method 9% to 13%) perpetrated violence in the following 12 months. Of these 131 outcomes, 55 were recorded in police data only, 43 in both police and EHR data, and 33 in EHR data only. Of all 1145 individuals assessed, 845 (74%) were offered follow-up under EIP services, of whom 85 (10%, 8% to 12%) perpetrated violence. Of the 300 individuals not followed up by EIP services, 46 (15%, 11% to 20%) perpetrated violence.

Prevalence of outcome and predictors in the validation cohort is presented in table 1 (see online supplemental table 4 for comparison with Swedish development sample).

**Table 1:** Distribution of violence outcomes and predictors in the total clinical cohort, and among those who did and did not perpetrate violence during 12-month follow-up

Predictor	Whole validation cohort (n=1145)	Individuals with at least one violent outcome during follow-up (n=131)	Individuals with no violent outcomes during follow-up (n=1014)	P value*
12-month violent outcome, n (%)	131 (11)	–	–	
Male, n (%)	687 (60)	109 (83)	578 (57)	**<0.001**
Age (years), mean (SD)	25 (10)	23 (9)	26 (10)	**<0.001**
Previous violent crime, n (%)	103 (9)	36 (27)	67 (7)	**<0.001**
Missing	12 (1)	4 (3)	8 (1)	
Previous drug misuse, n (%)	237 (21)	52 (40)	185 (18)	**<0.001**
Missing	3 (0)	1 (1)	2 (0)	
Previous alcohol misuse, n (%)	83 (7)	13 (10)	70 (7)	0.282
Missing	2 (0)	0 (0)	2 (0)	
Previous self-harm, n (%)	489 (43)	62 (47)	427 (42)	0.297
Missing	4 (0)	0 (0)	4 (0)	
Educational level, n (%)				**<0.001**
Lower secondary	348 (30)	63 (48)	285 (28)	
Upper secondary	312 (27)	37 (28)	275 (27)	
Post secondary	324 (28)	15 (11)	309 (30)	
Missing	161 (14)	16 (12)	145 (14)	
Parent drug or alcohol misuse, n (%)	134 (12)	26 (20)	108 (11)	**0.003**
Missing	137 (12)	19 (15)	118 (12)	
Parent violent crime, n (%)	34 (3)	10 (8)	24 (2)	**0.002**
Missing	489 (43)	70 (53)	419 (41)	
Sibling violent crime, n (%)	19 (2)	6 (5)	13 (1)	**0.016**
Missing	522 (46)	62 (47)	460 (45)	
Current episode inpatient, n (%)	214 (19)	16 (12)	198 (20)	0.057
Missing	0 (0)	0 (0)	0 (0)	
Recent antipsychotic treatment, n (%)	568 (50)	62 (47)	506 (50)	0.645
Missing	0 (0)	0 (0)	0 (0)	
Recent antidepressant treatment, n (%)	378 (33)	38 (29)	340 (34)	0.349
Missing	0 (0)	0 (0)	0 (0)	
Recent dependence treatment, n (%)	14 (1)	2 (2)	12 (1)	0.669
Missing	1 (0)	0 (0)	1 (0)	
Personal income category, n (%)				**<0.001**
Low	194 (17)	49 (37)	145 (14)	
Stable	941 (82)	82 (63)	859 (85)	
Missing	10 (1)	0 (0)	10 (1)	
Benefit recipient, n (%)	155 (14)	26 (20)	129 (13)	**0.035**
Missing	59 (5)	11 (8)	48 (5)	

Bold type face indicates p≤0.05.

*Refers to Χ^2^ test for differences in distribution of categorical variables among individuals with and without violent outcome, except age (two sample t-test) and recent dependence treatment (Fisher’s exact test).

### Missing data

Of the 16 predictors, 5 were missing at >1% (table 1). Missingness was consistent with missing at random (MAR) (online supplemental results 1). As per protocol, multiple imputation was used including all variables and outcome to enable MAR imputation.

### Model performance and updating

When the original OxMIV model was applied to the external validation dataset, discrimination as measured by pooled AUC was 0.75 (95% CI 0.71 to 0.80, online supplemental figure 2). The Brier score was 0.11.

**Figure 1:**
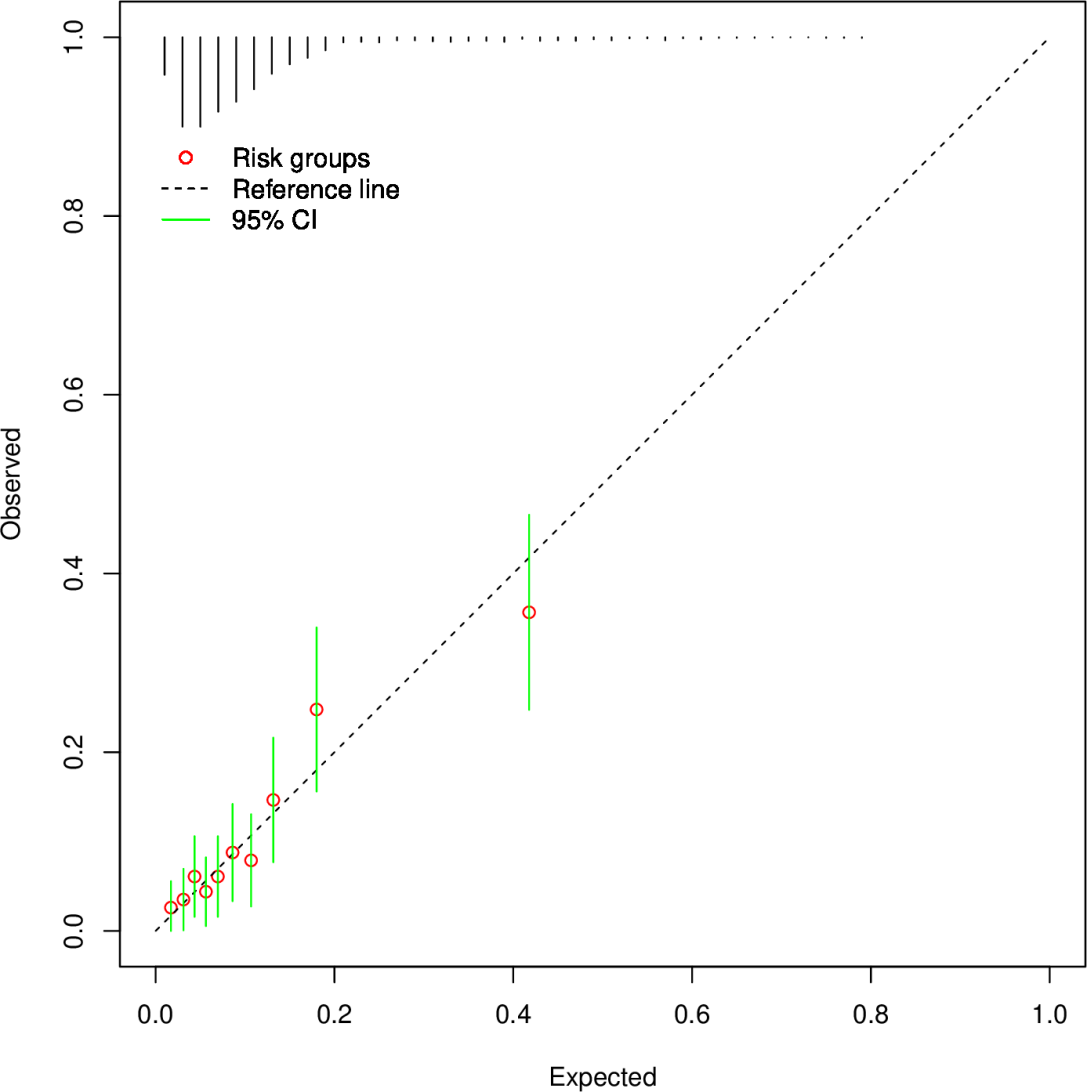
Calibration plot for updated OxMIV risk prediction model. Plotted with 10 risk groups in one imputed dataset for model with updated intercept. Histogram above legend represents distribution density of predicted risks within dataset.

Due to the different outcome definition and prevalence (11% in validation cohort vs 1% in development sample), there was miscalibration with the model underpredicting outcomes (online supplemental figure 3A). Expected/observed ratio was 0.18, calibration-in-the-large (CITL) was 2.00 (95% CI 1.80 to 2.19) and the calibration slope was 0.84 (0.66 to 1.03). Updating the intercept corrected the expected/observed ratio to 1.00 and CITL to 0.00 (−0.20 to 0.20) (figure 1). The Brier score improved to 0.09, suggesting good calibration. Discrimination and CITL were unchanged. Visual inspection of calibration plot showed residual miscalibration was largely at the upper end of predicted probabilities, which is less clinically important as this would likely be above any decision threshold. No substantial increase in performance was achieved by additionally using the calibration slope as a rescaling factor (online supplemental figure 3B, Brier score 0.09), and so the model with only the updated intercept was selected (online supplemental table 5).

### Decision threshold and clinical context

A range of relevant cut-off scores was considered for the updated model (see online supplemental table 6 for sensitivity, specificity, PPV and NPV across range). Optimal cut-off depends on the consequences of false negatives and the nature of any linked intervention. This balance was also considered by visualising the number of individuals identified as high or low risk, and the proportion correctly classified, at different cut-offs (online supplemental figure 4). Cut-offs of around 10%–12.5% provided most balance. At the 10% threshold, sensitivity of OxMIV was 71% (95% CI 63% to 80%), specificity 66% (63% to 69%), PPV 22% (19% to 24%) and NPV 95% (93% to 96%).

**Figure 2:**
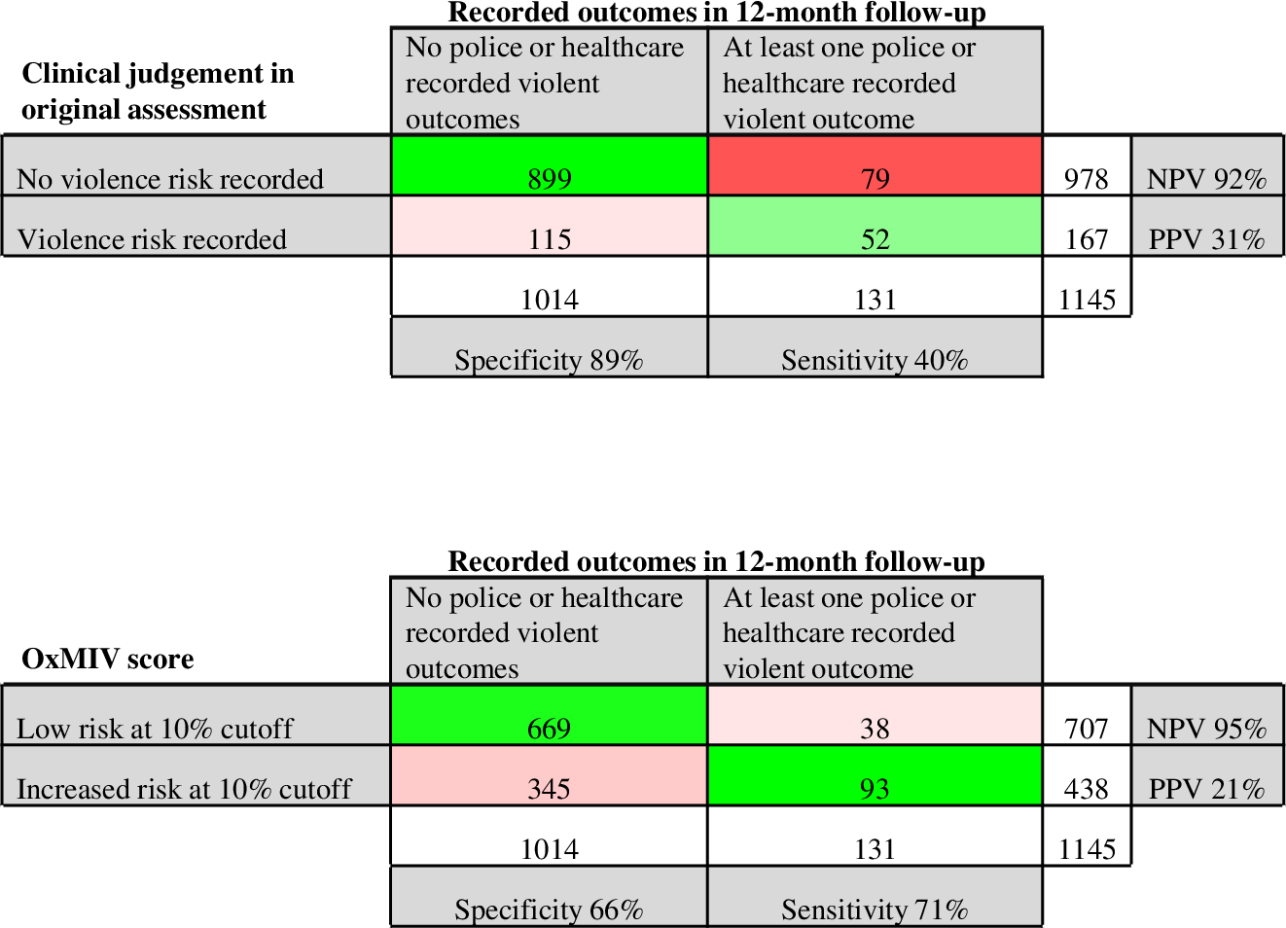
Misclassification matrix for OxMIV risk prediction model at 10% cutoff in one imputed dataset and for unstructured clinical assessment. Red cells indicate numbers of misclassified individuals (false negative and false positive), green cells indicate numbers of correctly classified individuals (true negative and true positive). Density of colour shading indicates relative proportion within columns, e.g. proportion of those with at least one violent outcome classified incorrectly or correctly. NPV, negative predictive value; PPV, positive predictive value.

The nature of the unstructured clinical assessments is also relevant to choosing the cut-off. Clinicians identified violence risk in 167 (15%) of all 1145 individuals assessed and in 52% of the 103 individuals who disclosed a previous violent offence. This approach had high specificity (89%) but low sensitivity (40%) for predicting violence (PPV 31%, NPV 92%). The 79 individuals misclassified as false negatives by unstructured assessment would have scored on average 18% using OxMIV. Figure 2 shows the 2×2 table of results (see online supplemental table 7 for a comparison of OxMIV rating and clinical judgement for individuals who did and did not perpetrate violence).

### Net benefit

**Figure 3:**
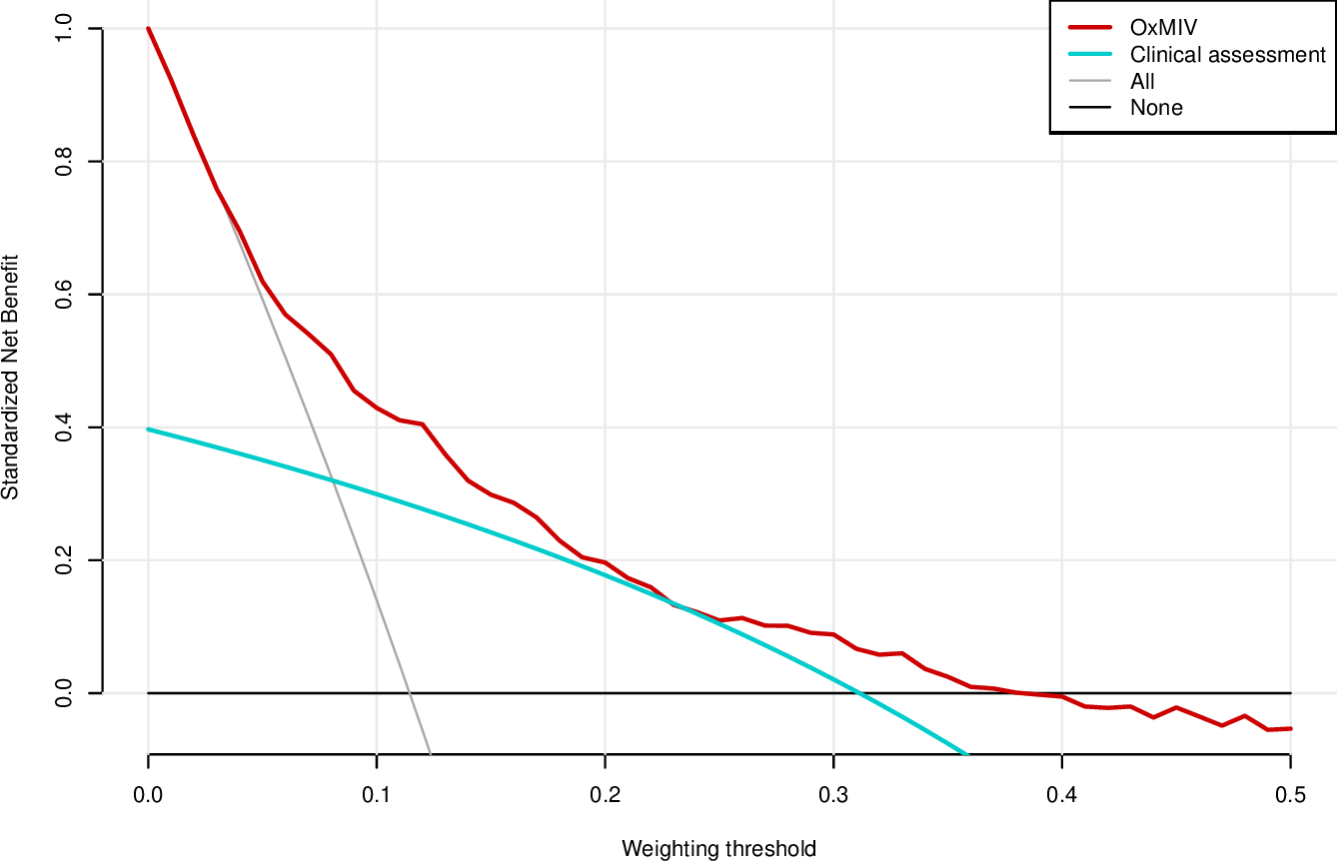
Decision curve analysis plot for OxMIV risk prediction model and for unstructured clinical assessment compared with default strategies of “treat all” or “treat none”. Value on x-axis indicates threshold for weighting of true positives and false positives. E.g. at 0.1, or odds of 1:9, missing a violent outcome is weighted as 9x worse than providing an unnecessary intervention. At 0.2, or odds of 1:4, missing a violent outcome is weighted as 4x worse than providing an unnecessary intervention, and so on for the range of preferences. Values toward zero indicate more concern about missing a violent outcome, and higher values indicate more concern about the harms of unnecessary intervention. The unit of net-benefit on y-axis is true-positives, standardized by dividing by the outcome proportion, i.e. proportion of patients with the outcome who would receive net benefit (after weighing up benefits of intervention and missing violent outcome).

Decision curve analysis (figure 3) suggested that, across a sensible range of preferences for what might be an acceptable ratio of false positives to true positives in the context of a given clinical intervention, allocating an intervention based on OxMIV can lead to higher benefit than simply offering that intervention to every patient or not offering it to any patients. This net benefit was higher than that of unstructured clinical assessment.

## Discussion

In this study of 1145 individuals accessing clinical services for early psychosis and followed up for 12 months, we assessed risk of violence perpetration with the published OxMIV model using predictors from electronic medical records, and ascertained violence outcomes from police and clinical data. Among the 1145 individuals assessed by EIP services, 131 (11%) perpetrated violence in the subsequent 12 months. OxMIV showed robust discrimination for prediction of violence (AUC 0.75, 95% CI 0.71 to 0.80). Calibration was also good and improved by updating the intercept to reflect the event rate for this population and outcome definition. Using a risk cut-off of 10%, sensitivity was 71% and specificity 66%. Decision curve analysis showed a net benefit of OxMIV over baseline strategies across a range of clinically realistic thresholds between 5% and 35%. Several implications follow for the potential clinical role of OxMIV in these services and for clinical prediction model research.

First, preventing violent outcomes in this population remains a clinically important goal. In this large clinical cohort, 11% perpetrated violence in the subsequent year, replicating previous studies.^6 7^ Two strong modifiable risk factors for violence, drug and alcohol misuse comorbidity^26^ were present in 21% and 7%, respectively, of the participants, which underlines the importance of identifying needs and reducing risk.

Second, we found that unstructured clinical judgement alone in assessing violence risk was suboptimal. The specificity of clinical judgement was 89%, but sensitivity was 40%; that is, the majority (60%) of individuals who went on to perpetrate violence did not have any risk in this domain identified by the assessing clinician. Among these individuals, risk factors for violence were objectively present. Thus, a key need is to improve the sensitivity of clinical assessments, and, to do so, using a 10% cut-off for classifying increased risk with OxMIV provides a good balance of over and under-identifying risk.

Third, the performance of OxMIV suggests that it can improve clinical assessments. Although comparing prediction models for diverse outcomes does not indicate clinical utility, the AUC in this external validation is comparable with risk tools adopted into clinical use elsewhere in medicine^17^ and with models developed in early psychosis populations, such as for transition to psychosis^12^ and cardiometabolic risk.^27^ It performs better than external validations of other violence risk assessment instruments (HCR-20 and VRAG)^15 28^ whose accuracy has been studied in individuals with psychosis (with AUCs of 0.71 and 0.69, respectively).^13^ These tools are lengthy and resource-intensive, and were studied in forensic psychiatric populations. OxMIV retained discriminative performance despite differences in casemix and prevalence of some predictors in the validation sample compared with the development population.^16^ This provides evidence for model transportability. Further, by not applying strict diagnostic criteria, the findings are applicable to real-world clinical practice.

Calibration was important as the event rate was higher than in the development study (as we used a broader measure than criminal conviction alone).^16^ The original model therefore underestimated observed outcomes. This is important if predictions in absolute terms are to be used. Re-estimating the model constant (ie, the intercept) adequately corrected this miscalibration, without altering predictor weighting. This step is essential to apply a prediction tool in a clinical setting, yet is frequently overlooked.^19^ The prevalence of violent outcomes in this study was in line with previous work,^7^ and so the application of the recalibrated model to this clinical setting is well supported. While the transportability of the model to different settings is also suggested by its performance, its use in different clinical populations requires further validation.

Regarding the externally validated model, some predictors may be correlated, but it is still important to retain predictors separately in the model for three reasons: first, clinical face validity; second, to make the model more robust if correlations were different in different participant settings, and, third, as the purpose of this study is external validation and altering the model would constitute redeveloping.

This study has also shown the feasibility of external validation using routine EHR data in a psychiatric setting, even when predictors are not recorded as structured fields. The scale at which this can be applied could be improved using techniques such as natural language processing.^29^ It was also shown that tightly defined register-based predictors can be successfully translated into pragmatic definitions for validation and clinical use.

By externally validating OxMIV in a clinically relevant setting, this study has undertaken the research necessary for adoption but this is done for a small minority of prediction tools.^12^ In addition, the current investigtion was adequately powered (including over 100 individuals with outcomes), and combined healthcare and police data to triangulate outcome information. Throughout, current evidence-based methods and reporting guidelines were followed,^20 24^ including the examination of calibration and approach to missing data.

The use of routine EHR data was necessary to allow data collection to occur offline from clinical practice. However, there are limitations in data not recorded for research purposes, which may vary in quality. For example, the clinical judgement comparator was collected from data recorded for clinical reasons: clinicians were not asked systematically to rate risk for the study, and judgements from routinely documented risk assessments were instead used. However, for the goal of comparing OxMIV with current real-world practice, which was a study aim, this is a valid comparator. Another limitation is that the predictor, past conviction, was likely under-reported. Although using official past conviction data would have improved accuracy, this information is not routinely available at assessment, and so it would not be clinically meaningful to use this for validation. The principle of data minimisation also precluded this approach. Further, the performance of OxMIV is examined as a standalone tool based on predictors from routine health records. Any additional value when used as an active part of the clinical assessment was not captured and requires further investigation.

Another limitation is that the investigation was based in two English regions. However, there is a clear model for EIP services which would support transferability to other services, which may require validation in other more geographically diverse populations. Some services for FEP impose a lower age limit than the study population’s upper bound of 65 years. However, given the wider evidence for the model’s transportability and the mean age of the study population of 25 years, results are likely generalisable to younger groups.

## Clinical implications

The potential clinical role of OxMIV can only be appraised with reference to a clinical decision pathway. This is challenging as this is not currently well standardised and likely to be multifaceted and individualised. However, decision curve analysis examines this by investigating a spectrum of preferences for whether minimising false negatives or false positives is preferable. OxMIV provided favourable net benefit across a clinically meaningful range of preferences. This indicates the potential value of OxMIV, but it is important to note that OxMIV would complement, rather than replace, unstructured clinical judgement. Currently, clinical judgement favours high specificity (ie, low false positives), which may be important for the current emphasis on crisis-based acute responses to violence risk.^26^ The key limitation of unstructured clinical judgement was low sensitivity of 40%, compared with 71% with OxMIV. Therefore, OxMIV could have an important and incremental role in improving sensitivity. There is emerging evidence that similar risk assessment tools can have a feasible clinical role, and this is a key target for future work.^30^ In theory, OxMIV could support clinicians to identify more needs around violence risk and offer additional follow-up services to a greater number of individuals for whom this might prevent a violent outcome, but importantly without offering this intervention to an unfeasibly large proportion, or to those for whom it is not needed on the basis of risk. This warrants evaluation in how it is used in practice, including for any unintended consequences such as over-reliance on tool outputs.

The PPV of OxMIV at the 10% cut-off was 22%, which means that one in five individuals classified as being at risk developed the outcome. Therefore, it is essential that linked interventions are non-harmful and should instead identify unmet needs and modifiable factors that can reduce risk to allocate resource accordingly. This could involve targeting co-occurring substance misuse, psychotic symptoms, facilitating engagement, or environmental factors such as accommodation. There are examples of a 10% decision threshold informing practice elsewhere in medicine to identify who is most likely to benefit from non-harmful risk-reduction strategies, such as whether to offer a statin for cardiovascular risk.

Two predictors (parental and sibling history of violent crime) were missing from the validation dataset in around half of cases. While the statistical approaches robustly account for this in validation, their presence in the clinical tool also merits practical consideration. One argument is that this may prompt clinicians to consider these factors more routinely, improving the overall quality of assessments. Whether this will be the case requires future evaluation. The web version of the OxMIV tool (https://oxrisk.com/oxmiv/) is already designed to be used when certain items are unknown, in which case the upper and lower bands of possible risk estimates are provided instead of a single point estimate.

### Conclusion

This study externally validated the OxMIV violence risk prediction model in a clinical population accessing services for early psychosis, using routine data, pragmatic and clinically operationalised predictor definitions, and an objective and clinically relevant measure of violence perpetration. The model showed good discriminative and overall performance and was well calibrated for relevant decision thresholds after updating to the outcome prevalence in this setting. Net benefit and measures of misclassification suggested benefits of OxMIV compared with current practice, with a role to improve the low sensitivity of unstructured clinical assessments. This could support clinicians to appropriately allocate interventions for reducing violence risk to those most likely to benefit.

## Data Availability

No data are available.
