# Supplementary material for: Assessing violence risk in first-episode psychosis: external validation, updating and net benefit of a prediction tool (OxMIV)
Source: BMJ Ment Health. 2023 Jun 14;26(1):e300634. doi: 10.1136/bmjment-2022-300634 (PMC10335427; doi:10.1136/bmjment-2022-300634)
Supplement: Supplementary data [file bmjment-2022-300634supp001.pdf]

Assessing violence risk in first episode psychosis: external validation, updating and net benefit of a prediction tool (OxMIV)

Daniel Whiting, Sue Mallett, Belinda Lennox and Seena Fazel

Supplemental Material

Whiting D, *et al. BMJ Ment Health* 2023; 26:e300634. doi: 10.1136/bmjment-2022-300634

Supplementary Table 1 3
Supplementary Table 2 5
Supplementary Table 3 12
Supplementary Methods 1 13
Supplementary Methods 2 17
Supplementary Methods 3 20
Supplementary Methods 4 22
Supplementary Figure 1 23
Supplementary Table 4 24
Supplementary Results 1 25
Supplementary Figure 2 26
Supplementary Figure 3 27
Supplementary Table 5 28
Supplementary Table 6 29
Supplementary Figure 4 30
Supplementary Table 7 32
Supplementary References 33

Supplementary Table 1

Tripod checklist.

Section/Topic		Checklist Item	Page
**Title and abstract**			
Title	1	Identify the study as developing and/or validating a multivariable prediction model, the target population, and the outcome to be predicted.	1
Abstract	2	Provide a summary of objectives, study design, setting, participants, sample size, predictors, outcome, statistical analysis, results, and conclusions.	2
**Introduction**			
Background and objectives	3a	Explain the medical context (including whether diagnostic or prognostic) and rationale for developing or validating the multivariable prediction model, including references to existing models.	5-7
	3b	Specify the objectives, including whether the study describes the development or validation of the model or both.	1-7
**Methods**			
Source of data	4a	Describe the study design or source of data (e.g., randomized trial, cohort, or registry data), separately for the development and validation data sets, if applicable.	7
	4b	Specify the key study dates, including start of accrual; end of accrual; and, if applicable, end of follow-up.	7
Participants	5a	Specify key elements of the study setting (e.g., primary care, secondary care, general population) including number and location of centres.	7
	5b	Describe eligibility criteria for participants.	7
	5c	Give details of treatments received, if relevant.	n/a
Outcome	6a	Clearly define the outcome that is predicted by the prediction model, including how and when assessed.	8
	6b	Report any actions to blind assessment of the outcome to be predicted.	8
Predictors	7a	Clearly define all predictors used in developing or validating the multivariable prediction model, including how and when they were measured.	8
	7b	Report any actions to blind assessment of predictors for the outcome and other predictors.	8
Sample size	8	Explain how the study size was arrived at.	9
Missing data	9	Describe how missing data were handled (e.g., complete-case analysis, single imputation, multiple imputation) with details of any imputation method.	9
Statistical analysis methods	10c	For validation, describe how the predictions were calculated.	9, Supplement
	10d	Specify all measures used to assess model performance and, if relevant, to compare multiple models.	9, Supplement
	10e	Describe any model updating (e.g., recalibration) arising from the validation, if done.	9, Supplement
Risk groups	11	Provide details on how risk groups were created, if done.	n/a
Development vs. validation	12	For validation, identify any differences from the development data in setting, eligibility criteria, outcome, and predictors.	Supplement
**Results**			
Participants	13a	Describe the flow of participants through the study, including the number of participants with and without the outcome and, if applicable, a summary of the follow-up time. A diagram may be helpful.	10, Supplement
	13b	Describe the characteristics of the participants (basic demographics, clinical features, available predictors), including the number of participants with missing data for predictors and outcome.	11
	13c	For validation, show a comparison with the development data of the distribution of important variables (demographics, predictors and outcome).	Supplement
Model performance	16	Report performance measures (with CIs) for the prediction model.	13
Model-updating	17	If done, report the results from any model updating (i.e., model specification, model performance).	12-14
**Discussion**			

Limitations	18	Discuss any limitations of the study (such as nonrepresentative sample, few events per predictor, missing data).	18-19
Interpretation	19a	For validation, discuss the results with reference to performance in the development data, and any other validation data.	17-18
	19b	Give an overall interpretation of the results, considering objectives, limitations, results from similar studies, and other relevant evidence.	16-20
Implications	20	Discuss the potential clinical use of the model and implications for future research.	19-20
**Other information**			
Supplementary information	21	Provide information about the availability of supplementary resources, such as study protocol, Web calculator, and data sets.	Through out
Funding	22	Give the source of funding and the role of the funders for the present study.	21

Whiting D, *et al. BMJ Ment Health* 2023; 26:e300634. doi: 10.1136/bmjment-2022-300634

Supplementary Table 2

Crime/Home Office codes for included violent offences.

001/01	1	Murder - victim one year of age or older
001/02	1	Murder - victim under one year of age
002/00	2	Attempt murder - victim aged under 1 year
002/00	2	Attempt murder - victim aged 1 year or over
019/02	19A	Sex - Sexual intercourse with woman / girl mental defective
019/17	19H	Sex - Rape of a boy under 13 - SOA 2003
019/18	19E	Sex - Attempt rape of a girl under 13 - SOA 2003
019/19	19H	Sex - Attempt rape of a boy under the age of 13 - SOA 2003
021/02	21	Sex - Offender of any age cause / incite a girl under 13 to engage in sexual activity - penetration - SOA 2003
021/12	21	Sex - Offender under 18 engage in penetrative sexual activity with girl under 13 - SOA 2003
021/13	21	Sex - Offender under 18 engage in penetrative sexual activity with boy under 13 - SOA 2003
021/14	21	Sex - Offender under 18 cause / incite a girl under 13 to engage in sexual activity - penetration - SOA 2003
070/01	70	Sex - Sexual activity with a mentally disordered male - penetration - SOA 2003
070/02	70	Sex - Sexual activity with a mentally disordered female - penetration - SOA 2003
070/05	70	Sex - Cause / incite sexual activity with a mentally disordered male - penetration - SOA 2003
070/06	70	Sex - Cause / incite sexual activity with a mentally disordered female - penetration - SOA 2003
071/02	71	Sex - Paying for the sexual services of a girl under 13 - penetration - SOA 2003
071/03	71	Sex - Paying for the sexual services of a boy under 13 - penetration - SOA 2003
005/14	05E	Possess firearm with intent to endanger life
005/15	05E	Possess shotgun with intent to endanger life
005/07	05E	Cause explosion with intent to burn / maim / grievous bodily harm etc
005/10	05E	Administer poison with intent to endanger life / inflict grievous bodily harm
005/13	05E	Do an act with intent to cause an explosion likely to endanger life / injure property
023/14	23	Sex - Adult sexual activity with a girl under 13 family member - penetration - SOA 2003
023/15	23	Sex - Adult sexual activity with a boy under 13 family member - penetration - SOA 2003
023/18	23	Sex - Adult incite sexual activity with a girl under 13 family member - penetration - SOA 2003
023/19	23	Sex - Adult incite sexual activity with a boy under 13 family member - penetration - SOA 2003
023/30	23	Sex - Offender under 18 sexual activity with a girl under 13 family member - penetration - SOA 2003
023/31	23	Sex - Offender under 18 sexual activity with a boy under 13 family member - penetration - SOA 2003
023/34	23	Sex - Offender under 18 incite sexual activity with a girl under 13 family member - penetration - SOA 2003
023/35	23	Sex - Offender under 18 incite sexual activity with a boy under 13 family member - penetration - SOA 2003
056/01	56A	Arson with intent to endanger life
071/12	71	Sex - Control a child under 13 involved in prostitution / pornography - SOA 2003
005/17	05E	Make use / attempt to make use of a firearm with intent to resist arrest
008/13	10A	Firearms - Possess firearm on arrest for Schedule 1 offence
008/13	10A	Firearms - Possess firearm while committing Schedule 1 offence
008/13	10A	Firearms - Possess imitation firearm on arrest for Schedule 1 offence
008/16	10A	Firearms - Have a firearm with intent to commit an indictable offence
008/16	10A	Firearms - Have an imitation firearm with intent to resist arrest
008/16	10A	Firearms - Have a firearm with intent to resist arrest
008/16	10A	Firearms - Have an imitation firearm with intent to commit an indictable offence
008/23	10A	Firearms - Possession of imitation firearm with intent to cause fear of violence

008/23	10A	Firearms - Firearm - possession with intent to cause fear of violence
008/24	10A	Firearms - Shotgun - possession with intent to cause fear of violence
019/07	19D	Sex - Rape a girl aged 13 / 14 / 15 - SOA 2003
019/08	19C	Sex - Rape a woman 16 years of age or over - SOA 2003
019/09	19G	Sex - Rape a male under 16
019/10	19F	Sex - Rape a man 16 or over - SOA 2003
019/11	19A	Sex - Attempted rape of a female under 16
019/12	19C	Sex - Attempt to rape a woman 16 or over - SOA 2003
019/13	19G	Sex - Attempted rape of a male under 16 years of age
019/14	19F	Sex - Attempt to rape a man aged 16 or over - SOA 2003
019/16	19E	Sex - Rape a girl under 13 - SOA 2003
021/04	21	Sex - Offender of any age cause / incite a boy under 13 to engage in sexual activity - penetration - SOA 2003
021/06	21	Sex - Offender 18 or over engage in penetrative sexual activity with girl under 13 - SOA 2003
021/07	21	Sex - Offender 18 or over engage in penetrative sexual activity with boy under 13 - SOA 2003
021/08	21	Sex - Offender 18 or over cause / incite a girl under 13 to engage in sexual activity - penetration - SOA 2003
021/09	21	Sex - Offender 18 or over cause / incite a boy under 13 to engage in sexual activity - penetration - SOA 2003
021/15	21	Sex - Offender under 18 cause / incite a boy under 13 to engage in sexual activity - penetration - SOA 2003
070/17	70	Sex - Care worker engage in sexual activity with mentally disordered male - penetration - SOA 2003
070/18	70	Sex - Care worker engage in sexual activity with mentally disordered female - penetration - SOA 2003
070/21	70	Sex - Care worker cause / incite sexual activity with mental disordered person - penetration - SOA 2003
071/14	71	Sex - Paying for the sexual services of a girl 13 to 15 - penetration - SOA 2003
071/15	71	Sex - Paying for the sexual services of a boy 13 to 15 - penetration - SOA 2003
005/01	05D	Assault - S18 - GBH grievous bodily harm with intent
005/01	05D	Assault - S18 - GBH cause grievous bodily harm with intent to resist / prevent arrest
005/04	05E	Attempt to choke / suffocate / strangle with intent
005/05	05E	Administer drug with intent to commit an indictable offence
008/03	10A	Firearms - Set a man trap with intent to inflict grievous bodily harm - Offences against the Person Act 1861
017/14	17B	Sex - Assault a boy under 13 by penetration with a part of your body / a thing - SOA 2003
020/04	20B	Sex - Assault a girl under 13 by penetration with a part of your body / a thing - SOA 2003
070/12	70	Sex - Sexual activity with mentally disordered person by inducement / threat / deception - no penetration - SOA 2003
070/14	70	Sex - Cause sexual activity with mentally disordered person by inducement / threat / deception - no penetration - SOA 2003
005/16	05E	Possess air weapon with intent to endanger life
023/04	23	Sex - Adult sexual activity with a girl 13 - 17 family member - penetration - SOA 2003
023/05	23	Sex - Adult sexual activity with a boy 13 - 17 family member - penetration - SOA 2003
023/08	23	Sex - Adult incite sexual activity with a family member - victim girl 13 to 17 - penetration - SOA 2003
023/09	23	Sex - Adult incite sexual activity with a family member - victim boy 13 to 17 - penetration - SOA 2003
023/32	23	Sex - Offender under 18 sexual activity with a girl 13 - 17 family member - penetration - SOA 2003
023/33	23	Sex - Offender under 18 sexual activity with a boy 13 - 17 family member - penetration - SOA 2003
023/36	23	Sex - Offender under 18 incite sexual activity with a girl 13 -17 family member - penetration - SOA 2003
023/37	23	Sex - Offender under 18 incite sexual activity with a boy 13 -17 family member - penetration - SOA 2003
004/01	42826	Manslaughter
008/13	10A	Firearms - Possess imitation firearm - committing Schedule 1 offence
028/02	28AB	Burglary dwelling - With intent to inflict GBH

Whiting D, *et al. BMJ Ment Health* 2023; 26:e300634. doi: 10.1136/bmjment-2022-300634

008/15	10A	Firearms - Possess air weapon while committing Schedule 1 offence
008/25	10A	Firearms - Air weapon - possession with intent to cause fear of violence
073/02	73	Sex - Sex offences - abuse position of trust - engage in sexual activity
073/01	73	Sex - Sex offences - abuse position of trust - have sexual intercourse
073/13	73	Sex - Adult abuse position of trust - sexual activity with a girl U.13 - SOA 2003
073/14	73	Sex - Adult abuse position of trust - sexual activity with a boy U.13 - SOA 2003
073/15	73	Sex - Adult abuse of position of trust - cause / incite sexual activity with girl U.13 - SOA 2003
073/16	73	Sex - Adult abuse of position of trust - cause / incite sexual activity with boy U.13 - SOA 2003
073/17	73	Sex - Adult abuse of position of trust - sexual activity in presence of child U.13 - SOA 2003
073/18	73	Sex - Adult abuse of position of trust - cause child under 13 to watch a sexual act - SOA 2003
005/07	05E	Cause taking / receiving of dangerous / noxious thing
005/07	05E	Send / deliver noxious thing with intent
008/04	08N	Cause bodily harm by wanton / furious driving
008/04	08N	Cause bodily harm by wilful neglect - vehicle
008/04	08N	Cause bodily harm by wilful misconduct
017/13	17A	Sex - Assault a male 13 or over by penetration with part of body / a thing - SOA 2003
020/03	20A	Sex - Assault a female 13 and over by penetration with part of body / a thing - SOA 2003
021/03	21	Sex - Offender of any age cause / incite a girl under 13 to engage in sexual activity - no penetration - SOA 2003
021/05	21	Sex - Offender of any age cause / incite a boy under 13 to engage in sexual activity - no penetration - SOA 2003
022/02	22A	Sex - Cause a female 13 or over to engage in a penetrative sexual activity - SOA 2003
022/03	22A	Sex - Cause a male 13 or over to engage in a penetrative sexual activity - SOA 2003
022/06	22B	Sex - Offender 18 or over engage in penetrative sexual activity with a girl 13 to 15 - SOA 2003
022/07	22B	Sex - Offender 18 or over engage in penetrative sexual activity with a boy 13 to 15 - SOA 2003
022/08	22B	Sex - Offender 18 or over cause / incite a girl 13 to 15 to engage in sexual activity - penetration - SOA 2003
022/09	22B	Sex - Offender 18 or over cause / incite a boy 13 to 15 to engage in sexual activity - penetration - SOA 2003
022/12	22B	Sex - Offender under 18 engage in penetrative sexual activity with a girl 13 to 15 - SOA 2003
022/13	22B	Sex - Offender under 18 engage in penetrative sexual activity with boy 13 - 15 SOA 2003
022/14	22B	Sex - Offender under 18 cause / incite a girl 13 to 15 to engage in sexual activity - penetration - SOA 2003
022/15	22B	Sex - Offender under 18 cause / incite a boy 13 to 15 to engage in sexual activity- penetration - SOA 2003
030/02	30A B	Burglary other than dwelling - With intent to inflict GBH
031/00	31	Aggravated burglary - other than dwelling
056/01	56A	Arson - recklessly endangering life
057/00/01	58A	Criminal damage to a dwelling - endangering life
057/00	58B	Criminal damage to a building other than a dwelling - endangering life
057/00	58C	Criminal damage to a vehicle - endangering life
057/00	58D	Criminal damage other - endangering life
059/11	59	Threaten to destroy / damage own property - endanger life
059/13	59	Have article with intent to destroy / damage property - endanger life
059/14	59	Make explosive substance for unlawful purpose
059/15	59	Possess explosive substance for unlawful purpose
071/11	71	Sex - Cause / incite a child under 13 to prostitution / pornography - SOA 2003
088/05	88C	Sex - Administer a substance with intent - SOA 2003
088/06	88C	Sex - Commit an offence with the intention of committing a relevant sexual offence - SOA 2003
088/07	88C	Sex - Knowingly / recklessly trespassed on premises with intent to commit a relevant sexual offence - SOA 2003

013/02	13	Child abduction - other person
036/01	36	Kidnap - common law
036/03	36	False imprisonment - common law
037/01	37/1	Aggravated vehicle taking - death caused by accident
008/59	08P	Racially / religiously aggravated wounding / GBH without intent
086/02	86	Make indecent photograph / pseudo-photograph of a child
086/02	86	Take an indecent photograph / pseudo-photograph of a child
088/01	88A	Sex - Adult meet girl under 16 following sexual grooming - SOA 2003
088/02	88A	Sex - Adult meet boy under 16 following sexual grooming - SOA 2003
004/02	42827	Infanticide
004/03	42828	Child destruction
029/00	29	Aggravated burglary - dwelling
1934/01/01	34A	Robbery - Business
1934/01/02	34B	Robbery - Personal
034/02	34A	Assault with intent to commit robbery - business
034/02	34B	Assault with intent to commit robbery - personal
035/00	35	Blackmail
066/03	66	Bomb hoax - place article with intent
066/04	66	Bomb hoax - communicate false information
071/09	71	Sex - Control a child 13 - 17 involved in prostitution / pornography - SOA 2003
071/10	71	Sex - Arrange / facilitate the prostitution / involvement in pornography of a child 13 - 17 - SOA 2003
071/13	71	Sex - Arrange / facilitate the prostitution / involvement in pornography of a child under 13 - SOA 2003

034/01		Robbery
008/02	08N	Cause administer poison with intent to injure / aggrieve / annoy
008/61	10C	Threaten with an offensive weapon in a public place
008/62	10D	Threaten person with blade or pointed article on school premises
008/64	10D	Threaten person with blade or pointed article in public place
008/65	08Q	Stalking - Involving fear of violence
008/66	08Q	Stalking - Involving serious alarm / distress
008/67	105A	Engage in controlling / coercive behaviour in an intimate / family relationship
005/24		Use of noxious substances or things to cause harm and intimidate
008/60	08P	Racially / religiously aggravated assault occasioning ABH
017/16	17B	Sex - Assault a boy under 13 by touching - SOA 2003
020/06	20B	Sex - Assault a girl under 13 by touching - SOA 2003
021/10	21	Sex - Offender 18 or over engage in sexual activity in presence of a child under 13 - SOA 2003
021/16	21	Sex - Offender under 18 engage in sexual activity in presence of a child under 13 - SOA 2003
021/18	21	Sex - Offender 18 or over engage in non penetrative sexual activity with girl under 13 - SOA 2003
021/19	21	Sex - Offender 18 or over engage in non penetrative sexual activity with boy under 13 - SOA 2003
021/2	21	Sex - Offender 18 or over cause / incite a girl under 13 to engage in sexual activity - no penetration - SOA

Whiting D, *et al. BMJ Ment Health* 2023; 26:e300634. doi: 10.1136/bmjment-2022-300634

0		2003
021/21	21	Sex - Offender 18 or over cause / incite a boy under 13 to engage in sexual activity - no penetration - SOA 2003
021/22	21	Sex - Offender under 18 engage in non penetrative sexual activity with a girl under 13 - SOA 2003
021/23	21	Sex - Offender under 18 engage in non penetrative sexual activity with a boy under 13 - SOA 2003
021/24	21	Sex - Offender under 18 cause / incite a girl under 13 to engage in sexual activity - no penetration - SOA 2003
021/25	21	Sex - Offender under 18 cause / incite a boy under 13 to engage in sexual activity - no penetration - SOA 2003
022/10	22B	Sex - Offender 18 or over engage in sexual activity in presence of a child 13 to 15 - SOA 2003
022/16	22B	Sex - Offender under 18 engage in sexual activity in presence of a child 13 to 15 - SOA 2003
070/03	70	Sex - Sexual activity with a mentally disordered male - no penetration - SOA 2003
070/04	70	Sex - Sexual activity with a mentally disordered female - no penetration - SOA 2003
070/07	70	Sex - Cause / incite sexual activity with a mentally disordered male - no penetration - SOA 2003
070/08	70	Sex - Cause / incite sexual activity with a mentally disordered female - no penetration - SOA 2003
070/19	70	Sex - Care worker engage in sexual activity with mentally disordered male - no penetration - SOA 2003
070/20	70	Sex - Care worker engage in sexual activity with mentally disordered female - no penetration - SOA 2003
070/22	70	Sex - Care worker cause / incite sexual activity with mentally disordered person - no penetration - SOA 2003
071/04	71	Sex - Paying for the sexual services of a girl 13 to 15 - no penetration - SOA 2003
071/04	71	Sex - Paying for the sexual services of a girl under 13 - no penetration - SOA 2003
071/05	71	Sex - Paying for the sexual services of a boy 13 to 15 - no penetration - SOA 2003
071/05	71	Sex - Paying for the sexual services of a boy under 13 - no penetration - SOA 2003
071/06	71	Sex - Paying for the sexual services of a girl aged 16 / 17 - SOA 2003
071/07	71	Sex - Paying for the sexual services of a boy aged 16 / 17 - SOA 2003
071/08	71	Sex - Cause / incite a child 13 - 17 to prostitution / pornography - SOA 2003
008/58	08M	Racially / religiously aggravated harassment with fear of violence
037/02	37/2	Aggravated vehicle taking - (initial taker) and dangerous driving
037/02	37/2	Aggravated vehicle taking - and vehicle damage of £5000 or over
008/30	08L	Public Order - S4 Harassment - put in fear of violence
008/55	09B	Racially / religiously aggravated intentional harassment / alarm / distress
008/56	08M	Racially / religiously aggravated harassment without violence
066/91	09B	Racially or religiously aggravated fear of violence
125/82	09B	Racially / religiously aggravated harassment / alarm / distress
195/12	08Q	Stalking - Pursue a course of conduct
008/01	08N	Assault - S20 - GBH Grievous bodily harm without intent
017/12	17A	Sex - Indecent assault on a man aged 16 or over
017/15	17A	Sex - Sexual assault on a male - SOA 2003

020/05	20A	Sex - Sexual assault on a female - SOA 2003
022/04	22A	Sex - Cause a female 13 or over to engage in a non penetrative sexual activity - SOA 2003
022/05	22A	Sex - Cause a male 13 or over to engage in a non penetrative sexual activity - SOA 2003
022/18	22B	Sex - Offender 18 or over engage in non penetrative sexual activity with girl 13 to 15 - SOA 2003
022/19	22B	Sex - Offender 18 or over engage in non penetrative sexual activity with boy 13 to 15 - SOA 2003
022/20	22B	Sex - Offender 18 or over cause / incite a girl 13 to 15 to engage in sexual activity - no penetration - SOA 2003
022/21	22B	Sex - Offender 18 or over cause / incite a boy 13 to 15 to engage in sexual activity - no penetration - SOA 2003
022/22	22B	Sex - Offender under 18 engage in non penetrative sexual activity with a girl 13 to 15 - SOA 2003
022/23	22B	Sex - Offender under 18 engage in non penetrative sexual activity with a boy 13 to 15 - SOA 2003
022/24	22B	Sex - Offender under 18 cause / incite a girl 13 to 15 to engage in sexual activity - no penetration - SOA 2003
022/25	22B	Sex - Offender under 18 cause / incite a boy 13 to 15 to engage in sexual activity - no penetration - SOA 2003
056/02	56B	Arson - not endangering life
003/01	03B	Threats to kill
008/06	08N	Assault - S47 - AOABH assault occasioning actual bodily harm
021/11	21	Sex - Offender 18 or over cause a child under 13 to watch / look at an image of sexual activity - SOA 2003
021/17	21	Sex - Offender under 18 cause a child under 13 to watch a sexual act - SOA 2003
022/11	22B	Sex - Offender 18 or over cause a child aged 13 to 15 to watch / look at an image of sexual activity - SOA 2003
022/17	22B	Sex - Offender under 18 cause a child 13 to 15 to watch a sexual act - SOA 2003
023/06	23	Sex - Offender under 18 sexual activity with a girl 13 - 17 family member - no penetration - SOA 2003
023/07	23	Sex - Offender under 18 sexual activity with a boy 13 - 17 family member - no penetration - SOA 2003
023/10	23	Sex - Offender under 18 incite sexual activity with a family member - victim girl 13 to 17 - no penetration - SOA 2003
023/11	23	Sex - Offender under 18 incite sexual activity with a family member - victim boy 13 to 17 - no penetration - SOA 2003
023/12	23	Sex - Offender 16 or over sexual activity with an adult relative over 18 - penetration - SOA 2003
023/13	23	Sex - Offender 16 or over consenting to sexual activity by an adult relative over 18 - penetration - SOA 2003
023/16	23	Sex - Offender under 18 sexual activity with a girl under 13 family member - no penetration - SOA 2003
023/17	23	Sex - Offender under 18 sexual activity with a boy under 13 family member - SOA 2003
023/20	23	Sex - Offender under 18 incite sexual activity with a girl under 13 family member - no penetration - SOA 2003
023/21	23	Sex - Offender under 18 incite sexual activity with a boy under 13 family member - no penetration - SOA 2003
023/22	23	Sex - Adult sexual activity with a girl 13 - 17 family member - no penetration - SOA 2003
023/23	23	Sex - Adult sexual activity with a boy 13 - 17 family member - no penetration - SOA 2003
023/24	23	Sex - Adult sexual activity with a girl under 13 family member - no penetration - SOA 2003
023/25	23	Sex - Adult sexual activity with a boy under 13 family member - no penetration - SOA 2003
023/26	23	Sex - Adult incite sexual activity with a family member - victim girl 13 to 17 - no penetration - SOA 2003
023/2	23	Sex - Adult incite sexual activity with a family member - victim boy 13 to 17 - no penetration - SOA 2003

7		
023/28	23	Sex - Adult incite sexual activity with a girl under 13 family member - no penetration - SOA 2003
023/29	23	Sex - Adult incite sexual activity with a boy under 13 family member - no penetration - SOA 2003
125/09	09A	Public Order - S4A sign etc to harass alarm distress
125/09	09A	Public Order - S4A words / behaviour to cause harassment / alarm / distress
125/11	09A	Public Order - S4 display sign etc intend unlawful violence
125/11	09A	Public Order - S4 words / behaviour - fear unlawful violence
125/68	08L	Harassment of a person in their home
008/20	08N	Assault with intent to resist arrest
104/23	104	Assault a constable in the execution of his / her duty
104/25	104	Assaulted a designated person / assistant - SOCA 2005
105/03	105A	Assault person assisting constable in execution of duty
105/04	105A	Assault prisoner custody officer
105/06	105A	Assault court security officer
105/08	105A	Assault designated / accredited person - Police Reform Act 2002
105/10	105A	Assault a traffic officer in the execution of his / her duty
105/01	105A	Assault - S39 - Common assault
125/12	09A	Public Order - S5 Display writing / sign - harassment alarm distress
125/12	09A	Public Order - S5 Use threatening words / behaviour to cause harassment alarm or distress
008/05		Assault with Injury

Supplementary Table 3

Predictor definitions pre-specified for extraction from electronic health records.

**Predictor *(code)***	**Definition**	**Data recorded**
Age *(Age)*	Age at time of assessment.	Age in years
Sex *(Sex)*	Sex at birth.	Male / female
Previous violent crime *(PrevViol)*	Any lifetime conviction for violence (assault with or without injury, homicide, robbery, arson, any sexual offence, illegal threats or intimidation). If not explicitly stated, but risk assessment identifies no history of risk towards others, recorded as 'No'.	Yes / No
Previous drug misuse *(PrevDrug)*	Lifetime diagnosis drug use disorder, from documented diagnosis, or indicated by past contact with drug rehabilitation/treatment services or detailed history.	Yes / No
Previous alcohol misuse *(PrevAlc)*	Lifetime diagnosis alcohol use disorder, from documented diagnosis, or indicated by past contact with alcohol rehabilitation/treatment services or detailed history.	Yes / No
Previous self-harm *(DSH)*	Lifetime episode of self-harm (any form, e.g. deliberate self-poisoning, cutting).	Yes / No
Highest education *(Education)*	Formal schooling: secondary (to age 16), upper secondary (to age 18), post-secondary (past 18).	Secondary / Upper secondary / Post-secondary
Parental drug or alcohol misuse *(ParentDrug)*	Parental lifetime diagnosis of drug or alcohol use disorder (definitions as above for personal history).	Yes / No
Parental violent crime *(ParentViol)*	Parental lifetime conviction for a violent offence (defined as above for personal history). History of incarceration taken as a proxy of violent offending.	Yes / No
Sibling violent crime *(SibViol)*	Sibling lifetime conviction for a violent offence (defined as above). History of incarceration taken as a proxy of violent offending.	Yes / No
Current episode *(Episode)*	Inpatient hospital admission or outpatient community patient at point of assessment.	Inpatient / Outpatient
Recent antipsychotic treatment *(Antipsych)*	Prescribed and taken any antipsychotic drug in 6 months before assessment.	Yes / No
Recent antidepressant treatment *(Antidep)*	Prescribed and taken any antidepressant drug in 6 months before assessment.	Yes / No
Recent dependence treatment *(Dependence)*	Any pharmacological strategy to treat dependence (e.g. replacement therapy such as methadone) prescribed and taken in 6 months before assessment.	Yes / No
Personal income*	Low: unemployed and/or inadequate financial situation	Low / Stable

(Incomecat)	with difficulty meeting basic daily living needs (food, accommodation etc.). Otherwise, 'stable'.	
Benefit recipient *(Benefit)*	Currently receiving social or disability benefits of any kind.	Yes / No

*For personal income, the development study used deciles [1], but stakeholder work indicated this was clinically not feasible and it was simplified.

Supplementary Methods 1

Choice of violent outcome measure

Officially registered violent outcomes such as arrest or conviction offer an advantage over measures that rely on ongoing contact with mental health services, as this engagement may be disrupted as a consequence of developing the outcome. Official outcomes also avoid limitations of self-report scales. Conviction is the most robust measure, however there are practical limitations in a psychiatric population. Police-recorded occurrences of an individual having been arrested, charged and/or a suspect for a violent offence were used as these objectively capture a significant incident that is stressful, disruptive and stigmatising, and also consume both police resource and healthcare resource for potential future care. Relevant offence codes were pre-specified (Supplementary Table 2). Codes for offences pertaining to possession alone of a weapon (i.e. without any associated threats or assaultive behaviour as defined above) were not included, as these can occur secondary to proactive policing measures. To be included, the date of the incident (rather than the date of arrest or charge) must have been within 12 months of the EIP assessment.

It is also well recognised that significant violent incidents may occur which do not lead to police contact. Typically these can occur during acute-phase illness, such as during an inpatient hospital admission, where normal criminal proceedings may not be pursued. Nevertheless, such incidents are of clinical importance. To avoid missing such outcomes therefore, police data was supplemented by a focussed review of the 12 month period in question in the EHR to identify any relevant documented incidents. The threshold of severity for this was pre-specified as involving a weapon or documented physical injury.

Whiting D, *et al. BMJ Ment Health* 2023; 26:e300634. doi: 10.1136/bmjment-2022-300634

Clinical judgement comparator

If the summary did not mention risk to others, or referred to risk as “low” (including synonyms such as “absent”, “not identified”, “nil significant”, “no concern”), this was recorded as “not increased”. Alternatively, if 1) there was any documentation that risk to others was “moderate” or “high” (including synonyms “elevated”, “escalating”, “concerning”, “acute”, “unsafe”), 2) the categorical option in the risk assessment template was marked “risk identified”, or 3) there were features within the management plan specifically addressing violence risk (for example documentation of safety advice given to a relative on how to respond in the event of aggression), then risk was recorded as “increased”.

Data extraction

If there was insufficient information to code a predictor, this was recorded “NA”. Data was extracted by one researcher (DW), and for a random sample of 20 individuals also blindly by a second researcher. Concordance was 90%-100% for all but three predictors (highest education [85%], sibling violent crime [85%] and parental violent crime [75%]). Discordance was around the threshold to code “NA” and was resolvable through discussion.

Missing data

The approach of multiple imputation by chained equations is the recommended approach in current statistical literature, and even allows external validation in cohorts that do not include all the original predictors [2]. Alternatives such as complete-case analysis are recognised as unnecessarily inefficient as they can disregard a substantial amount of available data and introduce unwanted selection bias because only patients with complete data can be included in analysis [3]. Further, entirely excluding predictors with missing data from models would revert the study to development of a new model, ignoring valuable predictive information from the original model development. The imputation model included all other predictors and

Whiting D, *et al. BMJ Ment Health* 2023; 26:e300634. doi: 10.1136/bmjment-2022-300634

the outcome. The plausibility of imputed datasets was checked by visually examining the distribution of predictors in observed and imputed data using stacked bar charts. The ability of pooling results from multiple imputations to give unbiased performance estimates and standard errors is well described [4].

Whiting D, *et al. BMJ Ment Health* 2023; 26:e300634. doi: 10.1136/bmjment-2022-300634

Supplementary Methods 2

Discrimination

Discrimination is the ability of a model to separate (i.e. order correctly) any random pair of individuals with and without the outcome, and was quantified with the AUC (area under the receiver operating characteristic [ROC] curve), which for a binary outcome is identical to the concordance or c-statistic.[5] An AUC of 1 represents perfect discrimination, whereas 0.5 indicates a model that performs no better than chance.

Calibration

Whilst discrimination is important, for a tool to be clinically usable it must also be adequately calibrated in external validation. Calibration is how well predictions match the observed data, i.e. do X% of individuals with a predicted risk of X% develop the outcome? Examining calibration in the current study is especially important, as the event rate was expected to differ from that in the development dataset.

Calibration was examined with 1) calibration-in-the-large (CITL) [6], which estimates the difference between the mean predicted outcomes and the mean observed outcomes where CITL<0 indicates that predicted probabilities are higher than observed proportions and vice versa, 2) the expected/observed ratio (E/O) [7], related to CITL but more intuitively summarising the ratio of the number predicted to have the outcome to the number observed to have the outcome, where 1 is perfect, 3) the calibration slope [6], where a calibration model is fitted and the slope coefficient indicates the degree of deviation from a perfect slope of 1 (<1 for an over-fitted model with predictions that are too extreme at low and high probabilities, and >1 for an under-fitted model), and 4) visual inspection of calibration plots, with predicted and observed probabilities plotted in decile risk groups.

Whiting D, *et al. BMJ Ment Health* 2023; 26:e300634. doi: 10.1136/bmjment-2022-300634

For overall model performance (encompassing both discrimination and calibration), the Brier score was calculated [5]. This is the squared difference between binary outcomes and predictions, with range from 0 to 1 with better scores being closer to 0.

Model updating

A stepwise approach was pre-specified [8]. First, baseline risk was adjusted by updating the model constant, bringing predicted and observed outcomes in line without affecting discrimination or predictor coefficients. Next, the calibration slope was used as a rescaling factor. The improvement in performance was examined to decide whether this rescaled model should be adopted in favour of the model with only the updated intercept (Supplementary Methods 3).

Clinical benefit

Measures of discrimination or calibration alone give limited information on clinical value [9,10]. This was addressed by examining net benefit with decision curve analysis (DCA) [11-13], informed by recent clarifications of its interpretation [14,15]. This specifies an "exchange rate" which is the number of false positive predictions that are acceptable to "treat" one true positive. This subjective preference depends on the relative harms of misclassification and is plotted across a range. The net benefit of OxMIV and unstructured clinical judgement were compared to baseline strategies of "treating none" or "treating all", as recommended for interpretation [14]. Intervening for all or no patients, irrespective of model results, may be reasonable clinical strategies and so, to justify clinical use, a model must be superior to both. For example, a poorly performing model may offer lower net benefit than offering a non-harmful intervention to all. For consistency and interpretation, for OxMIV, the exchange rate is the same as the cutoff for classifying "increased" risk.

Statistical analysis

Analyses were undertaken with R version 4.1 [16].

Supplementary Methods 3

Model updating and statistical analyses

The OxMIV model is expressed by the equation:

$$Y = A + B_1 * X_1 + B_2 * X_2 + \ldots$$

Where *Y* is the linear predictor which is then converted to a probability scale by the logit link function, *A* is a constant (representing the Y-axis intercept), *B* is the regression coefficient for each predictor variable, and *X* is the predictor variable. All corresponding values for the OxMIV model are published in the derivation study [1] (-3.7273 + 0.8400*Male – 0.0468*Age + 1.6149*Previous violent crime + …).

To update the intercept of the model, linear predictors were first calculated with the intercept term removed, and then a logistic model fitted with these new linear predictors as the offset term. To update both intercept and slope, a logistic model was fitted using the linear predictors with the intercept removed as the only predictor.

Confidence intervals were calculated with the exact, conservative Clopper Pearson method for sensitivity/specificity and asymptomatic logit intervals for predictive values [17,18]. Confidence intervals were also reported for the incidence of violence in the cohort using the Wilson method [19], as this may inform future work.

Decision curve analysis was undertaken using the rmda package [20]. Missing data was visualised with the packages Hmisc [21], mice [22] and VIM [23], and generalised linear models were fitted to examine associations with missingness of a predictor as a binary dependent variable. The mice [22] package was used for multiple imputation and subsequent pooled analyses, specifying logistic regression for imputation of binary data and a multinomial logit model for imputation of education (factor with >2 levels). Analyses of complete datasets were undertaken with the packages pROC [24], DescTools [25] and caret

[26], with performance measures reported where applicable as pooled point estimates with 95% confidence intervals derived from total variance across individual analyses of complete imputed datasets.

Whiting D, *et al. BMJ Ment Health* 2023; 26:e300634. doi: 10.1136/bmjment-2022-300634

Supplementary Methods 4

Public and patient involvement

Efforts were made to test the methodology of this study with a wide range of public and patient representatives. The primary focus of this public and patient involvement was to test the acceptability of the methodology of using routine health record data. Input also assisted in producing the patient-facing documentation, which was limited to a poster communicating the study activity and the mechanism for dissent that was displayed in relevant patient areas. Over the course of the different stages of design and approval, 17 individuals were involved. This included carers and individuals with personal experience of EIP and forensic mental health services. There was positivity around the potential clinical applicability of the work. The importance of the information security safeguards, particularly the interface with police, was highlighted by representatives, who were reassured by the formal frameworks in place.

Supplementary Figure 1

Flow chart of study processes.

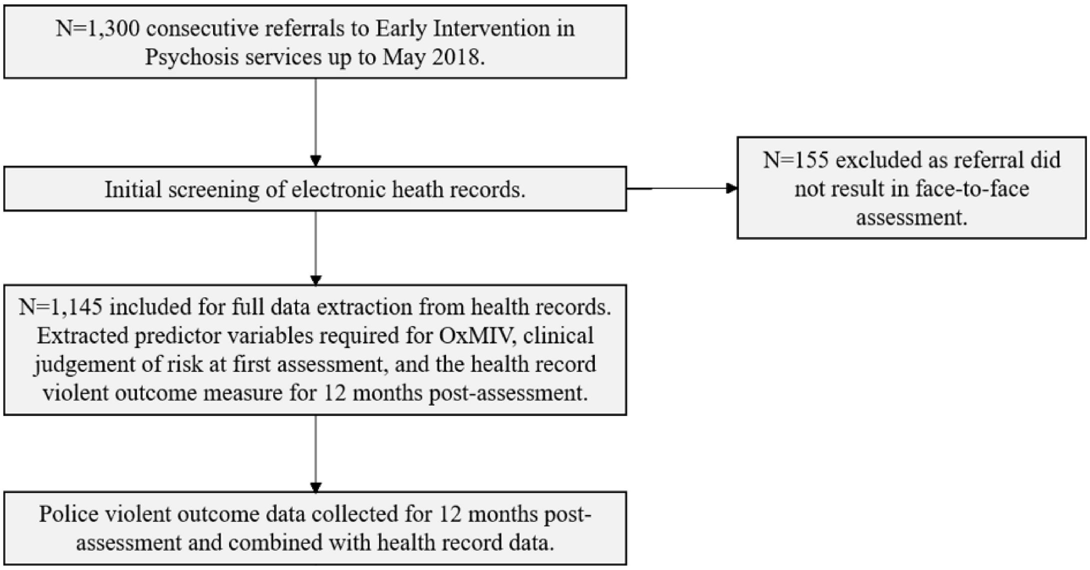

Supplementary Table 4

Distribution of outcome and predictors in validation cohort and Swedish development sample.

**Predictor**	**Validation cohort (n=1,145)**	**Development sample[1] (n=58,771)**
12 month violent outcome, n (%)	131(11%)	830 (1%)
Male, n (%)	687 (60%)	29,077 (49%)
Age (years), mean (SD)	25 (10)	44 (13)
Previous violent crime, n (%)	103 (9%)	9,212 (16%)
Missing	12 (1%)	
Previous drug misuse, n (%)	237 (21%)	7,123 (12%)
Missing	3 (0%)	
Previous alcohol misuse, n (%)	83 (7%)	8,897 (15%)
Missing	2 (0%)	
Previous self-harm, n (%)	489 (43%)	11,510 (20%)
Missing	4 (0%)	
Educational level, n (%)		
Lower secondary	348 (30%)	17,814/50,752 (35%)
Upper secondary	312 (27%)	26,449/50,752 (52%)
Postsecondary	324 (28%)	6,489/50,752 (13%)
Missing	161 (14%)	
Parent drug or alcohol misuse, n (%)	134 (12%)	5,214/47,957 (11%)
Missing	137 (12%)	
Parent violent crime, n (%)	34 (3%)	3,203/47,957 (7%)
Missing	489 (43%)	
Sibling violent crime, n (%)	19 (2%)	4,028 (7%)
Missing	522 (46%)	
Current episode inpatient, n (%)	214 (19%)	18,160 (31%)
Missing	0 (0%)	
Recent antipsychotic treatment, n (%)	568 (50%)	18,401/34,039 (54%)
Missing	0 (0%)	
Recent antidepressant treatment, n (%)	378 (33%)	13,255/34,039 (39%)
Missing	0 (0%)	
Recent dependence treatment, n (%)	14 (1%)	1,030/34,039 (3%)
Missing	1 (0%)	
Personal income category, n (%)		
Low	194 (17%)	5,444/57,867 (9%) (first decile)
Stable	941 (82%)	
Missing	10 (1%)	
Benefit recipient, n (%)	155 (14%)	37,210/57,876 (64%)
Missing	59 (5%)	

Whiting D, *et al. BMJ Ment Health* 2023; 26:e300634. doi: 10.1136/bmjment-2022-300634

Supplementary Results 1

Missing data

In 950 cases (83%), two or fewer of the 16 predictors were missing, and 471 (41%) were complete cases with no missing data. Examining the patterns of missingness among predictors showed that the family history items tended to be missing together; the most frequent overlap was for sibling violence and parental violence, which were both missing in 32% of cases. Clinically, it is plausible that the missingness of these two family history items depended on the level of detail of the assessment, rather than the missing value itself (i.e. a missing at random [MAR] pattern). Further, it would be expected that family history items would be less complete in older individuals (an observed variable), and more complete in individuals whose reference episode is a hospital admission (also an observed variable), as this typically means a fuller admission summary is documented with mandatory components. Margin plots showing the distribution of age when each of four predictors were either observed or missing (sibling violence, parental violence, parental drug and alcohol use and education) were in line with individuals with unobserved predictors tending to be older, in keeping with MAR. Generalised linear models showed that missingness of education and the three family history items was positively associated with age (i.e. older individuals were more likely to have these predictors missing, likely due to not being assessed to the same level of detail as younger persons for these factors), violating the missing completely at random (MCAR) assumption but in keeping with MAR.

Whiting D, *et al. BMJ Ment Health* 2023; 26:e300634. doi: 10.1136/bmjment-2022-300634

Supplementary Figure 2

Receiver Operating Characteristic (ROC) plot for 20 imputed datasets. Pooled Area Under the Curve (AUC) 0.753 (0.708-0.799).

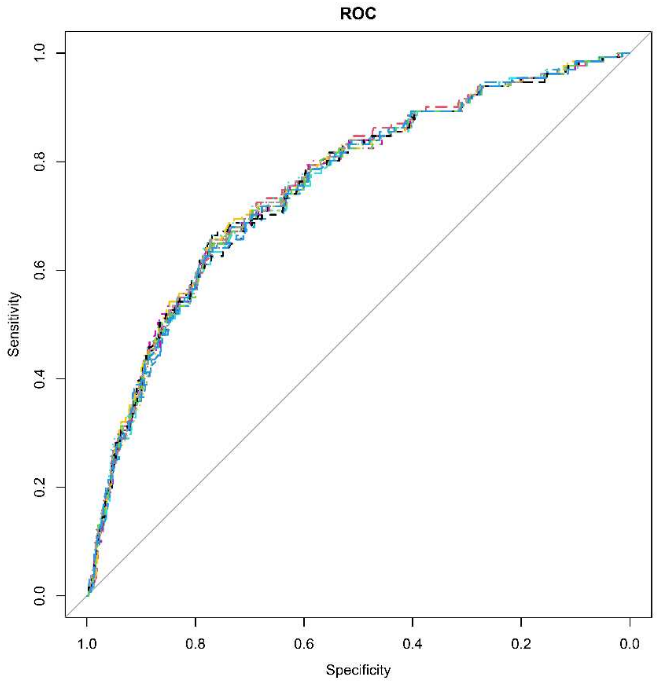

Supplementary Figure 3

Calibration plot with 10 risk groups, in an imputed dataset where OxMIV performs in the mid-range, for original model (a), and model with updated intercept and rescaled coefficients (b). Histogram above legend represents distribution density of predicted risks within dataset.

a)

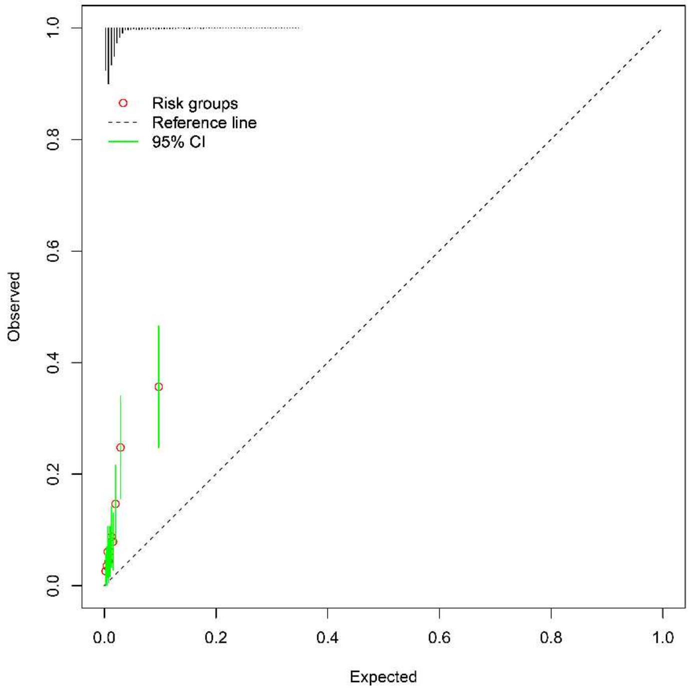

b)

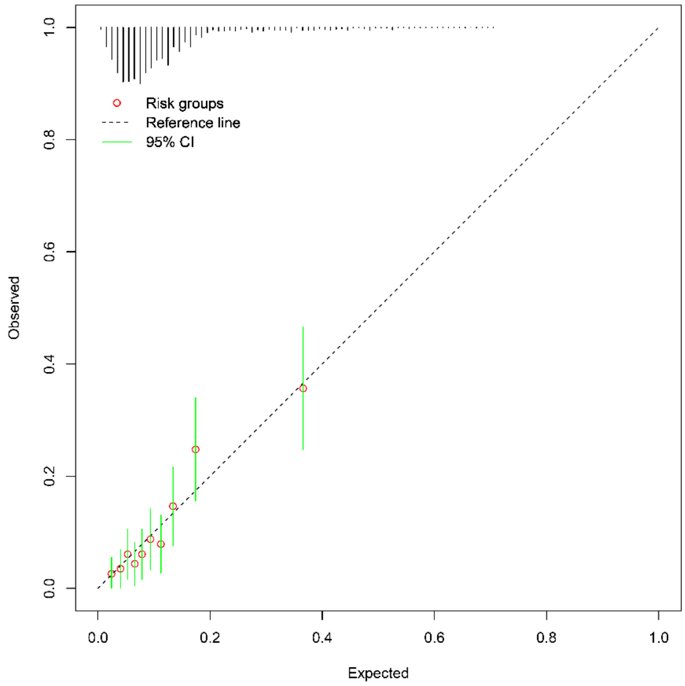

Supplementary Table 5

Externally validated model with updated intercept (constant) and p values from univariable analysis of external validation dataset.

**Predictor**	**Coefficient**	**p value in external validation cohort**
Constant	-1.7314	-
Male sex	0.8400	**<0.001**
Age	-0.0468	**<0.001**
Previous violent crime	1.6149	**<0.001**
Previous drug use	0.3737	**<0.001**
Previous alcohol use	0.5603	0.282
Previous self-harm	0.2049	0.297
Highest education		**<0.001**
Upper-secondary	-0.1264	
Post-secondary	-0.0703	
Parent drug or alcohol use	0.1044	**0.003**
Parent violent crime	0.1464	**0.002**
Sibling violent crime	-0.1076	**0.016**
Recent antipsychotic treatment	-0.4708	0.645
Recent antidepressant treatment	-0.2209	0.349
Recent dependence treatment	0.5783	0.669
Current episode inpatient	0.3143	0.057
Benefit recipient	0.3508	**0.035**
Income category stable	-0.1642	**<0.001**

Whiting D, *et al. BMJ Ment Health* 2023; 26:e300634. doi: 10.1136/bmjment-2022-300634

Supplementary Table 6

Paired misclassification measures for updated OxMIV model across range of cutoffs in one imputed dataset (note that final values in the main paper present pooled estimates from all imputed datasets).

	2.5%	5.0%	7.5%	10.0%	12.5%	15.0%	20.0%
Sensitivity (95% CI)	0.98 (0.93-1.0)	0.89 (0.83-0.94)	0.83 (0.76-0.89)	0.72 (0.63-0.79)	0.63 (0.54-0.71)	0.51 (0.42-0.60)	0.37 (0.28-0.46)
Specificity (95% CI)	0.11 (0.09-0.13)	0.33 (0.30-0.36)	0.53 (0.50-0.56)	0.66 (0.63-0.69)	0.78 (0.75-0.81)	0.84 (0.82-0.87)	0.91 (0.89-0.93)
PPV (95% CI)	0.12 (0.12-0.13)	0.15 (0.14-0.16)	0.19 (0.17-0.20)	0.22 (0.19-0.24)	0.27 (0.23-0.30)	0.30 (0.25-0.34)	0.35 (0.28-0.42)
NPV (95% CI)	0.97 (0.92-0.99)	0.96 (0.94-0.98)	0.96 (0.94-0.97)	0.95 (0.93-0.96)	0.94 (0.93-0.95)	0.93 (0.92-0.94)	0.92 (0.91-0.93)

Whiting D, *et al. BMJ Ment Health* 2023; 26:e300634. doi: 10.1136/bmjment-2022-300634

Supplementary Figure 4

a) Relationship between individuals identified as high risk and true positives across range of cutoffs from 0%-50%.

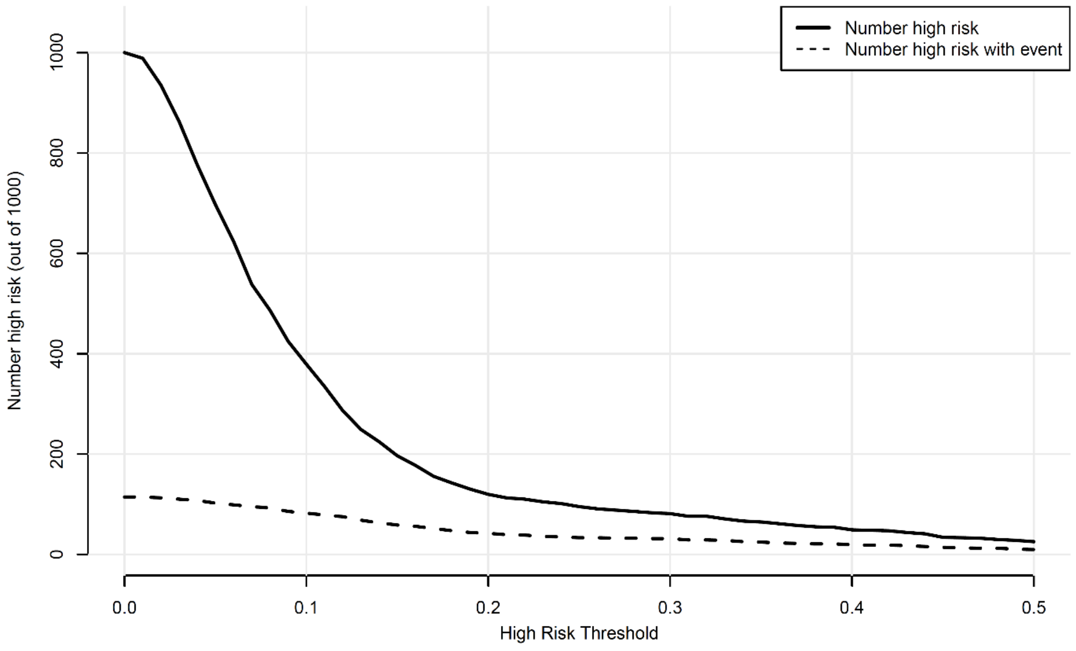

b) Relationship between individuals identified as low risk and true negatives across range of cutoffs from 0%-50%.

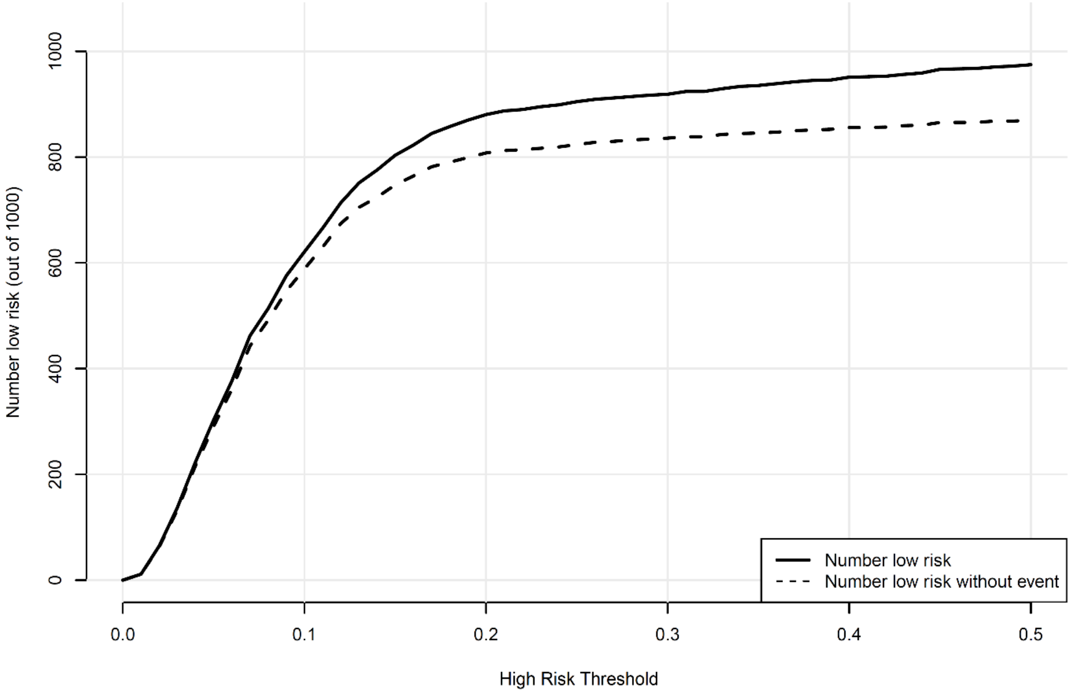

Supplementary Table 7

a) 2x2 table showing correspondence between categorical OxMIV rating at the 10% cutoff in one imputed dataset and the original clinical judgement made by the assessing clinician for the 131 individuals with at least one violent outcome during 12-month follow-up.

	**OxMIV Score**		
**Clinical judgement**	Increased risk at 10% cutoff	Low risk at 10% cutoff	
Risk identified at assessment	41	11	52
No risk identified at assessment	53	26	79
	94	37	131

b) 2x2 table showing correspondence between categorical OxMIV rating at the 10% cutoff in one imputed dataset and the original clinical judgement made by the assessing clinician for the 1,014 individuals with no violent outcomes during 12-month follow-up.

	**OxMIV Score**		
**Clinical judgement**	Increased risk at 10% cutoff	Low risk at 10% cutoff	
Risk identified at assessment	67	48	115
No risk identified at assessment	273	626	899
	340	674	1014

Whiting D, *et al. BMJ Ment Health* 2023; 26:e300634. doi: 10.1136/bmjment-2022-300634

Supplementary References

1. Fazel S, Wolf A, Larsson H, et al. Identification of low risk of violent crime in severe mental illness with a clinical prediction tool (Oxford Mental Illness and Violence tool [OxMIV]): a derivation and validation study. *Lancet Psychiatry.* 2017;4(6):461-468.
2. Held U, Kessels A, Garcia Aymerich J, et al. Methods for handling missing variables in risk prediction models. *Am J Epidemiol.* 2016;184(7):545-551.
3. Wolff RF, Moons KGM, Riley RD, Whiting PF, Westwood M, Collins GS, et al. PROBAST: a tool to assess risk of bias and applicability of prediction model studies: explanation and elaboration. *Ann Intern Med.* 2019;170(1):W1-w33.
4. Steyerberg EW. Missing values. In: Clinical prediction models: a practical approach to development, validation, and updating. Cham: Springer International Publishing. 2019;127-155.
5. Steyerberg EW, Vickers AJ, Cook NR, et al. Assessing the performance of prediction models: a framework for traditional and novel measures. *Epidemiology.* 2010;21(1):128-138.
6. Steyerberg EW. Evaluation of Performance. In: Clinical prediction models: a practical approach to development, validation, and updating. Cham: Springer International Publishing. 2019:277-308.
7. Riley RD, Ensor J, Snell KIE, et al. External validation of clinical prediction models using big datasets from e-health records or IPD meta-analysis: opportunities and challenges. *BMJ.* 2016;353:i3140
8. Steyerberg EW. Updating for a new setting. In: Clinical prediction models: a practical approach to development, validation, and updating. Cham: Springer International Publishing. 2019;399-429.
9. Vickers AJ, Cronin AM. Traditional statistical methods for evaluating prediction models are uninformative as to clinical value: towards a decision analytic framework. *Semin Oncol.* 2010;37(1):31-38.
10. Mallett S, Halligan S, Thompson M, Collins GS, Altman DG. Interpreting diagnostic accuracy studies for patient care. *BMJ : British Medical Journal.* 2012;345:e3999.
11. Vickers AJ, Elkin EB. Decision curve analysis: a novel method for evaluating prediction models. *Med Decis Making.* 2006;26(6):565-574.
12. Vickers AJ, Elkin EB. Decision curve analysis: a novel method for evaluating prediction models. *Med Decis Making.* 2006;26(6):565-574.
13. Steyerberg EW, Vickers AJ. Decision curve analysis: a discussion. *Med Decis Making.* 2008;28(1):146-149.
14. Vickers AJ, van Calster B, Steyerberg EW. A simple, step-by-step guide to interpreting decision curve analysis. *Diagn Progn Res.* 2019;3:18.
15. Van Calster B, Wynants L, Verbeek JFM, et al. Reporting and interpreting decision curve analysis: a guide for investigators. *Eur Urol.* 2018;74(6):796-804.
16. R Core Team. R: a language and environment for statistical computing. R Foundation for statistical computing, Vienna, Austria. URL www.R-project.org/.
17. Mercaldo ND, Lau KF, Zhou XH. Confidence intervals for predictive values with an emphasis to case-control studies. *Stat Med.* 2007;26(10):2170-2183.
18. Schaarschmidt F. bdpv: inference and design for predictive values in diagnostic tests. R package version 1.3. CRAN.R-project.org/package=bdpv.
19. Brown LD, Cai TT, DasGupta A. Interval estimation for a binomial proportion. *Stat Sci.* 2001;16(2):101-133, 133.
20. Brown M. rmda: risk model decision analysis. R package version 1.6 cran.r-project.org/web/packages/rmda/rmda.pdf.
21. Harrell F E Jr, with contributions from Dupont C and many others. Hmisc: Harrell miscellaneous. R package version 4.5-0. CRAN.R-project.org/package=Hmisc.
22. van Buuren S, Groothuis-Oudshoorn K. mice: multivariate imputation by chained equations in R. *J Stat Softw.* 2011;45(3):67.
23. Kowarik A, Templ M. Imputation with the R Package VIM. *J Stat Softw.* 2016;74(7):1 - 16.

24. Robin X, Turck N, Hainard A, et al. pROC: an open-source package for R and S+ to analyze and compare ROC curves. *BMC Bioinformatics.* 2011;12(1):77.
25. Signorell et mult. al. DescTools: tools for descriptive statistics. R package version 0.9.42.
26. Kuhn M. caret: classification and regression training. R package version 6.0-88. CRAN.R-project.org/package=caret.

Whiting D, *et al. BMJ Ment Health* 2023; 26:e300634. doi: 10.1136/bmjment-2022-300634
